# Correspondence between objective and perceived walking times to urban destinations: Influence of physical activity, neighbourhood walkability, and socio-demographics

**DOI:** 10.1186/1476-072X-11-43

**Published:** 2012-10-10

**Authors:** Bart Dewulf, Tijs Neutens, Delfien Van Dyck, Ilse de Bourdeaudhuij, Nico Van de Weghe

**Affiliations:** 1 Department of Geography, Ghent University, Krijgslaan 281, S8, B-9000, Ghent, Belgium; 2 Department of Movement and Sports Sciences, Ghent University, Watersportlaan 2, B-9000, Ghent, Belgium; 3 Research Foundation Flanders, Egmontstraat 5, B-1000, Brussels, Belgium

**Keywords:** Physical activity, Built environment, Geographical information system (GIS), Mental map, Walking time estimation

## Abstract

**Background:**

Doing regular physical activity has positive effects on health. Several environmental factors are identified as important correlates of physical activity. However, there seems to be a difference between perceived and objective measures of the environment. This study examines the influence of physical activity, neighbourhood walkability, and socio-demographic characteristics on the correspondence between self-reported and objectively measured walking time to urban destinations of adults in the city of Ghent (Belgium).

**Methods:**

Previously collected survey data was used from 1164 respondents in the city of Ghent who reported walking times to various closest destinations in the neighbourhood of residence. These were compared with corresponding walking times that were objectively measured through geographical information systems. Physical activity was recorded over a 7-day period using accelerometers. Neighbourhood walkability was assessed on the basis of residential density, connectivity, and land-use mix.

**Results:**

We observed a relatively poor agreement between objective and perceived walking times. Stronger agreements were noted amongst the most physically active group, while low-level walkers tended to overestimate walking time. Surprisingly, however, people residing in a low-walkable neighbourhood underestimated walking times more frequently relative to those in high-walkable neighbourhoods.

**Conclusions:**

Researchers investigating the influence of environmental attributes on physical activity behavior should thus be cautious when using only self-reported environmental data, since these are a priori influenced by physical activity levels and various socio-demographic factors.

## Background

### Introduction

Doing regular moderate-to-vigorous physical activity (MVPA) has several positive short- and long-term effects on health [1-4]. In 2008, approximately 31% of the global adult world population was not active enough to obtain these positive health effects [5]. Being insufficiently active is associated with an increased risk for several chronic diseases, like cardiovascular diseases, type 2 diabetes, obesity and some types of cancers [6-8]. Overall, being insufficiently active is related to premature deaths, resulting in heavy economic costs [6,8]. No changes in activity levels have been observed, and obesity rates and sedentary activities have increased during the last decade for example in North America and Australia, despite efforts that seek to encourage physical activity (PA) [9].

It is therefore important to develop insight in the correlates of PA and to develop a comprehensive population-based approach in promoting PA instead of an individual approach, which is the case nowadays [9-13]. Next to personal, cultural, and socio-economical factors, environmental attributes have been identified as important correlates of PA. A bourgeoning number of studies have offered compelling evidence that the physical environment influences people’s propensity to engage in physically active pursuits [13-19]. For example, Humpel et al. [20] found a positive relationship between accessibility and aesthetic attributes with PA in several reviewed articles. In another review article, Saelens et al. [14], for their part, identified 14 studies where an association between several neighbourhood attributes (e.g. accessibility, land use mix, access to public transport, and population density) and PA occurred. In a related study, Saelens et al. [15] concluded that people living in high walkable neighbourhoods in San Diego, California (US) engaged in approximately 52 more minutes of PA during a week compared to their counterparts living in low walkable neighbourhoods. Likewise, Owen et al. [16] reviewed 18 articles and observed that several environmental attributes (i.e. aesthetics, walking facilities, accessibility, and traffic perceptions) are linked with walking behavior.

However, these environmental attributes can be assessed in either an objective or perceived manner. Objective environmental attributes are measured using detailed georeferenced data by means of geographical information systems (GIS), while perceived attributes stem from self-reports in the form of surveys or questionnaires. Both types of attributes do not necessarily coincide and therefore may relate differently with physical activity behavior. For example, while objective availability of pertinent destinations in a neighborhood may be high, a person’s perceived availability can be low due to the fact that a person may not be aware of all feasible destinations in her/his neighborhood [21,22]. A decreased environmental awareness may in turn lead to a lower propensity to walk in that neighborhood, although the objective availability of destinations suggests otherwise. People process and store information about their environment according to their own attitudes, motivations, and preferences. These perceptions are not necessarily precise representations of the actual objective environment [23,24]. Incorporating both objective measures and perceptions of residents in research is important, as the impact of the objective environment on health depends on human perceptions, motivation, and deliberation [24].

In response to this potential discrepancy between the objective and perceived environment, several studies have scrutinized the concordance between objective and perceived environmental attributes, such as accessibility, walkability, dwelling density, street connectivity, land use mix, and retail density. Cerin et al. [25], for example, observed moderate to high correspondence between objective and perceived access to services, ease of walking, street connectivity, and walkability, whereas Ball et al. [26] found only a poor agreement between perceived and objective availability of PA facilities. Additionally, Ball et al. [26] noticed a greater mismatch between objective and perceived availability of PA facilities for less active people. However, they only examined whether or not certain facilities lie within a buffer zone around respondents’ location of residence (i.e. availability), but did not investigate distances to these facilities (i.e. accessibility). In a similar vein, Gebel et al. [27] observed a fair overall agreement between objective and perceived measures for dwelling density, intersection density, land use mix, and retail area. They found that less active people are more likely to misperceive the walkability of their neighbourhood. The reason for this is that more active people walk more in their neighbourhood, resulting in a better awareness of the environment [28-30]. Gebel et al. [27] additionally found that male, higher educated, normal weighted, older people from high walkable neighbourhoods make more correct estimations of environmental attributes.

### Related work

Instead of examining previously mentioned environmental attributes, this paper studies the agreement between objective and perceived walking times from respondents’ residences to different locations. Only few studies examined the agreement between objective and perceived walking distances/times to date. Jilcott et al. [31] and Macintyre et al. [32], for example, observed a fair agreement between objective and perceived walking distances to parks, gyms, and schools, while McCormack et al. [33] and Lackey & Kaczynski [34] noticed only a poor agreement for these destinations. Besides general agreement, some studies also studied the degree of underestimation or overestimation. In both Jilcott et al. [31] and McCormack et al. [33], it was concluded that on average the perceived walking distance to several destinations is greater than the objective walking distance, presumably because people can be unaware of the existence certain close facilities. An overestimation of walking distance in self-reported data was also identified in many earlier studies [30,35-37].

The agreement between objective and perceived walking distances/times can depend on several factors, with PA having the strongest influence. Because of greater environmental exposure and concomitant locational awareness, active people not only have a better perception of the previously mentioned attributes such as walkability and connectivity, but they can also make more accurate estimates of walking distances/times [33,34]. Regarding shops, McCormack et al. [33] found that less active people overestimate the distance in comparison to their active counterparts. Looking at distances to parks, Lackey & Kaczynski [34] concluded that people who did at least some park-based PA can more accurately appraise walking distances, since they experience more intimate and slow speed interaction with the places resulting in better distance estimates [20,22,28,29,38]. However, reasoning also works the other way around: people with a good mental map of the environment might be more likely to be physically active, because they are more familiar with the local environment. However, to date, no literature was found to substantiate this direction of causation. Next to PA, other factors have also been tested. McCormack et al. [33] observed, for instance, that people from high walkable neighbourhoods in Adelaide (Australia) overestimated distances to several destinations. Also, it has been pointed out that people overestimate short and well-known routes and underestimate long and less-known routes [30,35,39,40]. Considering other socio-demographic variables, Lackey & Kaczynski [34] concluded that younger, high educated, and normal weighted people have higher odds of achieving a match between objective and perceived proximity to parks in Ontario (Canada).

This study seeks to add to the knowledge base surrounding the above discussion by bringing additional evidence to the fore that sheds new light on the differential effects of objective and perceived access to urban destinations on physical activity.

The first objective is to analyze the agreement between objective and perceived walking times for residents from the city of Ghent. This is done by comparing objective and perceived walking times from one’s residence to different facilities (e.g. bakery, restaurant, and swimming pool etc.). The second objective is to test whether or not this agreement depends on PA, neighbourhood walkability, gender, educational level, body mass index (BMI), and age. It will be determined whether the degree of underestimation or overestimation differs depending on the previously mentioned factors.

## Methods

### Participants and procedures

For this study, data was used from the Belgian Environmental Physical Activity Study (BEPAS), conducted between May 2007 and September 2008 in the city of Ghent (237,000 inhabitants, 156.18 km^2^, and 1,468 inhabitants/km^2^). An equal number of respondents were selected from 24 neighbourhoods, containing one to five adjacent statistical sectors. Statistical sectors are the smallest units for which demographical data is available. An equal proportion of neighbourhoods with high/low walkability (explained later) and high/low socio-economic status (SES) based on neighbourhood level income data was selected. This means that six neighbourhoods are high walkable/high SES, six are high walkable/low SES, six are low walkable/high SES, and six are low walkable/low SES. From each neighbourhood, 250 adults age 18–66 were randomly selected by the Public Service of Ghent. Two to six days after receiving an informational letter on the study, home visits were made to potential participants, until 50 participants in each neighbourhood agreed to compete in the study. Overall response rate was 58% (2069 possible participants found at home, of which 1200 were willing to compete). From these participants, 1,164 had datasets that could be used for this study. For a more detailed description of the procedures, the reader is referred to Van Dyck et al. [19].

### Measures

#### Perceived walking times

As part of a questionnaire assessing perceived environmental attributes in the neighbourhood (Neighbourhood Environmental Walkability Scale (NEWS)), respondents were asked to estimate walking times to various closest destinations: supermarket, bakery, butchery, clothes shop, post office, library, primary school, restaurant, bank, video shop, pharmacy, bus or tram stop, and swimming pool [14,25,41]. Response options included: 1-5min, 5-10min, 11-20min, 21-30min, and more than 30 min. Previously, it has been shown that this NEWS survey has strong reliability and validity [15]. In the remainder of the paper, this self-reported walking time will be referred to as the perceived walking time.

#### Objective walking times

Objective walking times to the closest facilities were calculated in ArcGIS 9.0™ using Network Analyst. This was done by calculating the shortest route from residents’ home locations (available from the survey) to different types of destinations (available from a large and detailed inventory from 2009 of urban destinations in the city of Ghent). A GIS street network layer of routes available for walking, including exclusive pedestrian paths, is used in this analysis. These walkable paths are exported from the Large-Scale Reference Base (in Dutch: GRB, Grootschalig Referentiebestand), which is a highly accurate (20 cm) geographical database with information about various characteristics of roads, buildings, railroads, water areas, and parcels and will soon be available for the whole of Flanders [42]. Computed shortest distances were transformed into walking times by dividing them by an average walking speed. Average walking speeds were differentiated by gender and age according to Bohannon [43]. Bohannon calculated these average comfortable speeds from 230 healthy individuals (see Table 1). Following McCormack et al. [33], 0.3 km/h was subtracted from the average speeds to correct for stopping at crossings and for turning. The calculated walking times were then grouped into the same categories as those available in the NEWS questionnaire (i.e. 1-5min, 6-10min, 11-20min, 21-30min, and >30min) in order to be able to compare these to the self-reported walking times.

**Table 1:** Average corrected walking speeds

***Age group***	***Male average corrected walking speed (km/h)***	***Female average corrected walking speed (km/h)***
18-30	4.71	4.77
31-40	4.95	4.79
41-50	4.96	4.71
51-60	4.71	4.72
61-70	4.59	4.37
>70	4.49	4.28

Based on the results of Bohannon [43], from a sample of 230 individuals and corrected according to the results of McCormack et al. [33].

#### Physical activity (PA)

In order to estimate the level of PA, participants were asked to wear an accelerometer (model 7164, Computer Science Application) for seven consecutive days. Accelerometers have proven to be a valid and reliable instrument for PA assessment in adults [44,45]. The accelerometers were set to measure the number of accelerations per minute. 1,952 to 5,724 accelerations per minute correspond with moderate PA, and >5,724 accelerations per minute correspond with vigorous PA [46]. Only data from participants with at least 10 h wear time for at least four days (including at least one weekend day) were included in the study. From the raw data, the average time of moderate-to-vigorous physical activity (MVPA) per day was calculated. To dichotomize this variable, the health norm was used, which is recommended by several organizations [2,8,47,48]. The American College of Sports Medicine also recommends this health norm [49]. It stipulates that adults with at least 30 min of MVPA per day, for at least five days per week are physically active enough to take advantage of health benefits. Adults who do not reach this threshold are considered physically insufficiently active.

#### Neighbourhood walkability

Neighbourhood walkability indicates ‘the extent to which characteristics of the built environment and land use are conducive to walking for leisure, exercise or recreation, to access services, or to travel to work’ [50]. Using a GIS, a neighbourhood walkability index was constructed on the basis of three environmental variables: street connectivity, residential density, and land use mix [14]. These environmental variables were obtained from the Service for Environmental Planning in Ghent. A more detailed description on how this neighbourhood walkability is calculated can be found in Van Dyck et al. [19].

#### Socio-demographic variables

From the survey, different personal and socio-demographic factors were obtained, including gender, educational level (higher education (i.e. college or university degree) or not), BMI (≥25: overweight or <25: normal weight), and age (dichotomized to 18–45 and >45 years).

### Analyses

#### Objective 1: Agreement between objective and perceived walking time

The first objective of this study is to examine the degree of agreement between objective and perceived walking times. To test whether the difference between objective and perceived walking times is significant, a Wilcoxon *t*-test was used. This was done for the separate destinations as well as for all destinations together. To calculate average (objective and perceived) walking times, the time categories were transferred to the mean value. Also the total proportion of underestimations, correct estimations, and overestimations was calculated for all destinations using cross tabs. Correct estimations occur when the perceived walking time class is the same as the objective walking time class. Underestimations and overestimations occur when the perceived walking time class is respectively lower and higher than the objective walking time class.

#### Objective 2: Relation between different factors (PA, neighbourhood walkability, gender, educational level, BMI, and age) and degree of agreement

The second objective of this study is to assess the relation between different factors and the degree of agreement between objective and perceived walking times. To assess the odds of achieving a match (i.e. objective and perceived walking time are in the same category) in relation to the different factors, a logistic regression model was constructed. In this logistic regression, the odds ratios of making a correct estimation were calculated, depending on the different factors. If the 95% confidence interval does not include the null value 1, the selected part of the respondents (depending on the factor) has higher/lower odds of achieving a match. For factors found to be significant, the proportion of people making an underestimation, correct estimation or overestimation was calculated again, but now for the two ends of the factor (e.g. active and insufficiently active people) for all destinations separately. The proportion of underestimations, correct estimations, and overestimations were also calculated for the other factors, for all destinations combined. The logistic regression was repeated to assess the odds of making an underestimation or overestimation.

## Results

### Descriptive statistics

In Table 2 the descriptive statistics of the study sample are given. The sample contains slightly more active than insufficiently active respondents. The number of people from high and low walkable neighbourhoods is almost equal. There are more females than males in the sample. The majority of the sample has a higher education and normal weight and there are approximately 10% more 18–45 year olds in comparison with 46–66 year olds.

**Table 2:** Descriptive statistics (N=1164)

***Characteristic***	***N***	***%***
PA^a^		
Insufficiently active	560	48.1
Active	604	51.9
Gender		
Male	558	47.9
Female	606	52.1
Educational level		
No higher education	450	38.7
Higher education	701	60.2
Missing	13	1.1
BMI^b^		
Normal weight	705	60.6
Overweight	418	35.9
Missing	41	3.5
Age		
18-45 years	646	55.5
46-66 years	518	44.5
Neighbourhood walkability		
Low	583	50.1
High	581	49.9

^a^ Physical activity.

^b^ Body mass index.

### Objective 1: Agreement between objective and perceived walking time

The percentage of participants with available perceived walking time data was calculated (Table 3). It can be inferred that these percentages are very high and vary only slightly between different destinations. Table 3 also shows the average objective and perceived walking times for all destinations combined and for all destinations separately. Clothes shops, post offices, libraries, video shops, and swimming pools are on average located farthest from the respondents, while bus or tram stops tend to be present closest to the respondents’ home location. In addition, Table 3 shows the average difference between perceived and objective walking times. It is clear from Wilcoxon’s test that for all but two destinations (i.e. post office and library), participants significantly overestimate the objective walking time. The absolute average difference is greatest for supermarkets, clothes shops, and restaurants. For post offices there is a significant underestimation of objective walking time.

**Table 3:** Average objective and perceived walking times, average differences, underestimations, correct estimations, and overestimations

***Destination***	***Respondents for whom perceived walking time was available (%)***	Average objective walking time (min)*	Average perceived walking time (min)*	***Average difference (min)***	***Underestimation (%)***	***Correct estimation (%)***	***Overestimation (%)***
All destinations	97.2	13	16	3**	13.9	52.2	33.9
Bus or tram stop	99.5	3	4	1**	2.6	83.2	14.2
Restaurant	98.9	6	13	7**	6.9	39.1	54.0
Primary school	98.0	8	12	4**	12.0	47.2	40.8
Bakery	99.2	8	9	1**	15.3	63.8	20.9
Pharmacy	99.1	8	10	2**	5.8	66.5	27.7
Supermarket	99.3	9	17	8**	7.1	27.7	65.2
Butchery	99.3	10	11	1**	14.3	55.2	30.5
Bank	99.2	10	14	4**	8.7	50.0	41.3
Clothes shop	98.5	16	22	6**	10.9	42.8	46.3
Video shop	97.2	18	20	2**	18.4	56.0	25.6
Post office	99.3	21	20	−1**	28.3	50.3	21.4
Library	98.8	24	23	−1	29.5	45.5	25.0
Swimming pool	98.7	24	26	2**	20.4	51.5	28.1

* Time category midpoints were used to calculated average values.

** p<0.001 from Wilcoxon *t*-test.

From this average difference, no further information about the proportions of underestimations, correct estimations or overestimations can be deduced. Therefore, cross tabs were made with the objective and perceived walking times from all destinations combined and separately. From these cross tabs, the total proportion of people making an underestimation, correct estimation and overestimation were calculated. This can be found in the final three columns of Table 3. On average, for all destinations combined, 52.2% of the respondents made a correct estimation, 13.9% made an underestimation, and 33.9% made an overestimation. The largest proportion of correct estimations is found for bakeries, butcheries, video shops, pharmacies, and bus or tram stops. Most underestimations are found for post offices, libraries, and swimming pools. These are typically the destinations, which are generally located farthest away from the location of residence. Overestimations are most prominent for supermarkets, clothes shops, and restaurants.

### Objective 2: Relation between different factors (PA, neighbourhood walkability, gender, educational level, BMI, age) and degree of agreement

Table 4 depicts the results of a logistic regression, performed to assess the relation between different factors and the degree of agreement between objective and perceived walking times. PA is the only significant predictor of the degree of agreement (OR=1.138), suggesting that active people have higher odds of achieving a match between objective and perceived walking times.

**Table 4:** Logistic regression to test the relation between different factors and the degree of agreement between objective and perceived walking times

***Factor (concerning category)***	***Odds Ratio***	***95% Confidence Interval***
PA^a^ (active)	1.138*	1.068-1.214
Gender (female)	0.972	0.911-1.036
Educational level (higher education)	1.010	0.945-1.078
BMI^b^ (overweight)	0.965	0.902-1.032
Age (>45 years)	1.054	0.989-1.124
Neighbourhood walkability (high)	0.992	0.931-1.058

* p<0.05 from the logistic regression.

^a^ Physical activity.

^b^ Body mass index.

**Figure 1:**
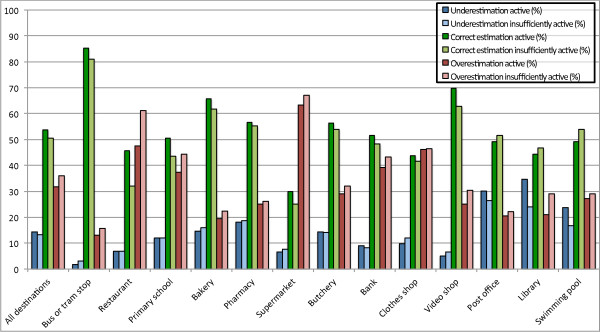
Proportion of underestimations, correct estimations, and overestimations, for active and insufficiently active people.

The logistic regression only tells us something about the degree of agreement, but it does not give any information about whether walking times are underestimated or overestimated. Hence, Figure 1 shows the proportion of both active and insufficiently active people making an underestimation, correct estimation, and overestimation. For all destinations combined, it can be observed that active people make more correct estimations than insufficiently active people, which aligns with the results from the logistic regression. In addition, active people make more underestimations than insufficiently active people and insufficiently active people make more overestimations than active people.

For all destinations separately (except for post offices, libraries, primary schools, and swimming pools) active people make more correct estimations than insufficiently active people. For butcheries, post offices, libraries, banks, and swimming pools active people make more underestimations than insufficiently active people. The overall result is that for all destinations insufficiently active respondents make more overestimations than active people.

As can be seen in Table 4, no significant results were obtained for the other factors from the logistic regression. But since the logistic regression only estimates the odds ratios of achieving a match, the proportion of underestimations, correct estimations, and overestimations were additionally calculated for the other factors for all destinations combined. The results are summarized in Table 5. Significant results from the additional logistic regression are marked with an *. It can be inferred that male, normal weight, younger people make significantly more underestimations and significantly less overestimations than their female, overweight, older counterparts. Also, people from a low walkable neighbourhood make almost 5% more underestimations than people from a high walkable neighbourhood. In addition, people from a high walkable neighbourhood make almost 5% more overestimations than people from a low walkable neighbourhood.

**Table 5:** Proportion of underestimations, correct estimations, and overestimations, for different factors for all destinations combined

***Factor***		***Underestimation (%)***	***Correct estimation (%)***	***Overestimation (%)***
PA^a^	Insufficiently active	13.2*	50.6*	36.1*
	Active	14.4*	53.7*	31.9*
Gender	Male	14.4*	52.6	32.9*
	Female	13.3*	51.9	34.9*
Educational level	No higher education	13.6	52.3	34.1
	Higher education	14.0	52.2	33.8
BMI^b^	Normal weight	14.5*	52.5	33.0*
	Overweight	13.0*	51.6	35.4*
Age	<=45j	15.0*	51.6	33.4
	>45j	12.3*	53.0	34.7
Neighbourhood walkability	Low	16.2*	52.3	31.5*
	High	11.5*	52.1	36.4*

^a^ Physical activity.

^b^ Body mass index.

* p<0,05 from the logistic regression.

## Discussion

### Objective 1: Agreement between objective and perceived walking time

The first objective of the study was to examine the agreement between objective and perceived walking times to various closest destinations. This agreement was found to be relatively poor: on average 52.2% of the respondents made a correct estimation. This finding aligns with Macintyre et al. [32] and Jilcott et al. [31], who respectively found a correspondence of 62.0% and 60.9%. However, the observed agreement strongly differs from that of Lackey & Kaczynski [34] (17.9%) and McCormack et al. [33] (11.4%). However, it ought to be noted that it is difficult to compare with the studies of Macintyre et al. [32] and Lackey & Kaczynski [34] since they have only studied perceived and objective access to parks by verifying whether there is a park within 750 m from one’s residence or not. Furthermore, in our study, 33.9% of the respondents tended to overestimate the objective walking time. This general overestimation was also found in earlier studies as mentioned in the specific literature review and may be explained by the fact that people can be unaware of certain close facilities [31].

More specifically, when separate destinations are considered, the furthest destinations (swimming pools, libraries, post offices, and video shops) have the largest proportion of underestimations. This is similar to the results of McCormack et al. [33], where the two farthest destinations (libraries and post offices) also represented the largest amount of underestimations. Also in accordance with McCormack et al. [33], the walking time to supermarkets is overestimated most. Proffitt et al. [51] mention a possible explanation for this: it has been shown that carrying heavy bags requires more physical effort, which results in distance overestimations. Additionally, people often go shopping by car to prevent carrying heavy bags or to make sure that frozen goods do not melt. The use of motorized transport causes less interaction with the environment [22], resulting in more overestimations [28-30]. Another possible explanation for the overestimation of walking time to supermarkets is that small (often foreign) shops are also included in the data, although people may not patronize these shops as frequently as larger shops. Walking times to destinations that are most common (bakeries, butcheries, pharmacies, and bus or tram stops) are most often estimated correct. Also walking times to video shops and swimming pools are estimated rather well, probably because only few of these facilities exist which are therefore well known.

### Objective 2: Relation between different factors (PA, neighbourhood walkability, gender, educational level, BMI, age) and degree of agreement

As mentioned in the introduction, it has previously been shown that active people can better estimate walking distances/time because of their greater exposure and awareness resulting from more intense interaction with the environment [20,28,29,33,34,38]. The logistic regression carried out in this paper showed that active people actually have higher odds (OR=1.138) of achieving a match between objective and perceived walking distances. Detailed analyses showed that active people make 3.1% more correct estimations than insufficiently active people. While McCormack et al. [33] observed that insufficiently active people overestimate only the distance to shops, this paper found that insufficiently active people overestimate walking times to all destinations. More specifically, insufficiently active people make 4.2% more overestimations than active people. Additionally, active people make 1.2% more underestimations than insufficiently active people.

Since an earlier study in Ghent showed that people from high walkable neighbourhoods tend to be more active than people from low walkable neighbourhoods [19], it was expected that people from high walkable neighbourhoods would make more underestimations, whereas people from low walkable neighbourhoods would make more overestimations. However, our results showed that there is almost no difference in the proportion of correct estimations between high and low walkable neighbourhoods and that residents of low walkable neighbourhoods make more underestimations, while those of high walkable neighbourhoods make more overestimations. There may be two explanations for this. First, the higher degree of overestimations of distance can be explained by the presence of more intersections in high walkable neighbourhoods [28,52,53]. Second, routes to destinations in high walkable neighbourhoods are often relatively short and it has been shown earlier that short and well-known routes are more often overestimated, whereas long and unknown routes are more often underestimated [30,35,39,40].

For the other demographic variables (gender, educational level, BMI, and age) no significant results were found in Macintyre et al. [32] and Lackey & Kaczynski [34]. This coincides with the results of this study, since these factors had no significant influence on the odds of making a correct estimation. However, results of this paper show that male, normal weighted, younger people make more underestimations and less overestimations, than female, overweighted, older people. These results are as expected, because male, normal weighted, and younger people are more active [54].

### Study strengths and limitations

This study has several strengths. First, it is to our knowledge the first study in European mainland about the effect of physical activity behavior on travel time estimations and on associations between objective and perceived walking distances to destinations. Other studies concerning the relationship between the environment and PA are mainly North American and Australian. Previous studies were conducted, among others, in South Carolina (US; [38]), North Carolina (US; [31]), Glasgow (UK; [32]), Adelaide (Australia; [33]), and Ontario (Canada; [34]). Second, the sample of 1,164 respondents used in this study is larger than that of many other similar studies: 86 in McCormack et al. [33], 199 in Jilcott et al. [31], 574 in Lackey & Kaczynski [34], and 658 in Macintyre et al. [32]. Only in Kirtland et al. [38] a similar number of participants (1,112) were studied. Third, more types of destinations are taken into consideration: 13 in contrast with 1 to 9 in the previously mentioned studies. Fourth, as in Jilcott et al. [31], this study uses accelerometer data to estimate PA, which is more objective compared to the self-reported data used in many previous studies including Kirtland et al. [31], McCormack et al. [33], Macintyre et al. [32], and Lackey & Kaczynski [34]. Fifth, body mass index (BMI) has been taken up as an explanatory variable in this study, which is not the case in the other five similar studies (ibid.). Sixth, in contrast to prior work, walking time is used instead of walking distance. The advantage of this is that walking speeds, and thus walking time – in contrast to walking distance – can be differentiated according to gender and age [43].

Apart from the many advantages our study has over similar studies, there are also limitations. First, it is possible that people do not know the closest facility of a particular type simply because they are unaware of it. Incorporating the time that respondents have lived in their neighbourhood might help to gain insights in this effect. Second, the questionnaire (NEWS) used for this study uses predefined categories to estimate the perceived walking time to various closest destinations. The reason for this is to minimize errors, because it can be hard to estimate walking times with a precision of one minute. However, because of these categories, short objective walking times can not be underestimated and long objective walking times can not be overestimated. Third, in choosing routes, people are often driven by sense of safety, attractiveness and complexity of the environment, and emotional responses [15,16,30,36,55], and therefore do not necessarily take the shortest route possible. Future studies comparing objective and perceived walking times should therefore include the actual routes, possibly making use of the GPS technology, and compare these with the objective and perceived shortest routes.

## Conclusions

While in the past several studies used perceived walking times or distances as a substitute for actual walking times as a measure for access to different facilities (e.g. [56,57]), this study has shown that these perceived walking times/distances are often an overestimation of the objective walking times/distances. Future studies should keep this poor correspondence in mind, as well as the fact that when only using self-reported walking times, the results can be influenced by physical activity and other variables. In general, people overestimate walking times, but physically insufficiently active people in particular make even more overestimations, probably because of their inadequate mental map resulting from lower interaction and experience with their residential neighbourhood. By overestimating walking times, people can be discouraged to walk and might end up being insufficiently active. These vicious circle effects should make policy makers aware that in order to promote physical activity, one should not only look at the objective neighborhood characteristics but also at how people of socio-demographic segments and with different PA levels may perceive these. It is important for policymakers to appreciate that by influencing people’s perception, one can change PA behavior without adjusting the environment itself.

## Competing interests

The author(s) declare that they have no competing interests.

## Authors’ contributions

BD carried out the main research and drafted the manuscript. TN participated in the study design and helped to draft the manuscript. DVD helped with the statistical analyses. IdB participated in the study design. NVdW coordinated the study. All authors reviewed the manuscript and approved the final version.
